# The Seagrass Methylome Is Associated With Variation in Photosynthetic Performance Among Clonal Shoots

**DOI:** 10.3389/fpls.2020.571646

**Published:** 2020-09-04

**Authors:** Alexander Jueterbock, Christoffer Boström, James A. Coyer, Jeanine L. Olsen, Martina Kopp, Anusha K. S. Dhanasiri, Irina Smolina, Sophie Arnaud-Haond, Yves Van de Peer, Galice Hoarau

**Affiliations:** ^1^Algal and Microbial Biotechnology Division, Faculty of Biosciences and Aquaculture, Nord University, Bodø, Norway; ^2^Marine Molecular Ecology Group, Faculty of Biosciences and Aquaculture, Nord University, Bodø, Norway; ^3^Environmental and Marine Biology, Åbo Akademi University, Åbo, Finland; ^4^Shoals Marine Laboratory, University of New Hampshire, Durham, NH, United States; ^5^Ecological Genetics-Genomics Group, Groningen Institute of Evolutionary Life Sciences, University of Groningen, Groningen, Netherlands; ^6^UMR MARBEC, Université de Montpellier, Ifremer, IRD, CNRS, Sète, France; ^7^Department of Plant Biotechnology and Bioinformatics, Ghent University, Ghent, Belgium; ^8^Bioinformatics and Systems Biology, VIB Center for Plant Systems Biology, Ghent, Belgium; ^9^Center for Microbial Ecology and Genomics, Department of Biochemistry, Genetics and Microbiology, University of Pretoria, Pretoria, South Africa

**Keywords:** DNA methylation, ecological epigenetics, clonality, heat stress, seagrass, *Zostera marina* (eelgrass)

## Abstract

Evolutionary theory predicts that clonal organisms are more susceptible to extinction than sexually reproducing organisms, due to low genetic variation and slow rates of evolution. In agreement, conservation management considers genetic variation as the ultimate measure of a population’s ability to survive over time. However, clonal plants are among the oldest living organisms on our planet. Here, we test the hypothesis that clonal seagrass meadows display epigenetic variation that complements genetic variation as a source of phenotypic variation. In a clonal meadow of the seagrass *Zostera marina*, we characterized DNA methylation among 42 shoots. We also sequenced the whole genome of 10 shoots to correlate methylation patterns with photosynthetic performance under exposure to and recovery from 27°C, while controlling for somatic mutations. Here, we show for the first time that clonal seagrass shoots display DNA methylation variation that is independent from underlying genetic variation, and associated with variation in photosynthetic performance under experimental conditions. It remains unknown to what degree this association could be influenced by epigenetic responses to transplantation-related stress, given that the methylomes showed a strong shift under acclimation to laboratory conditions. The lack of untreated control samples in the heat stress experiment did not allow us to distinguish methylome shifts induced by acclimation from such induced by heat stress. Notwithstanding, the co-variation in DNA methylation and photosynthetic performance may be linked *via* gene expression because methylation patterns varied in functionally relevant genes involved in photosynthesis, and in the repair and prevention of heat-induced protein damage. While genotypic diversity has been shown to enhance stress resilience in seagrass meadows, we suggest that epigenetic variation plays a similar role in meadows dominated by a single genotype. Consequently, conservation management of clonal plants should consider epigenetic variation as indicator of resilience and stability.

## Introduction

Genetic variation is considered key to long-term survival of populations (Bijlsma and Loeschcke, 2012), and is recognized as a key for the conservation and restoration of biological diversity (Lande, 1988; Spielman et al., 2004; Laikre et al., 2009). In contrast, lack of genetic variation is regarded as an evolutionary dead-end (Lynch and Lande, 1998) but clonal growth challenges the expected relationship between genetic diversity and long-term survival. For example, conservation management follows the rough guideline that within a population >1,000 genetically different individuals must mate randomly to avoid inbreeding depression, and retain evolutionary potential (Frankham et al., 2014). Yet, roughly 40% of all plants can reproduce asexually (Tiffney and Niklas, 1985), mostly by clonal growth where parental genotypes (genets) grow vegetative modules (ramets) that often remain connected *via* underground stolons or rhizomes.

Although clones benefit from resource sharing, niche specialization, and rapid vegetative growth (Liu et al., 2016), they are predicted to survive only for short periods, and in stable environments (Silvertown, 2008). Asexual reproduction is assumed to lead to slow rates of genetic evolution, and the lack of DNA repair mechanisms afforded by meiosis (Muller’s ratchet, mutational meltdown) (Muller, 1964; Gabriel et al., 1993; Lynch et al., 1993). Despite asexual reproduction, many of our most important crops are clones (McKey et al., 2010), including banana, garlic, hops, potatoes, and turmeric, as well as many of the earth’s most invasive and oldest plants. For example, genets of Palmer’s oak (*Quercus palmeri*) and seagrass (*Posidonia oceanica*) are estimated to be older than 10,000 years (May et al., 2009; Arnaud-Haond et al., 2012). Thus, long-term survival appears not to rely solely on sexual reproduction.

Somatic mutations can create a certain level of genetic diversity, and may explain some evolutionary potential of clonal organisms (Whitham and Slobodchikoff, 1981; Loxdale et al., 2003; Lushai et al., 2003; Reusch and Boström, 2011). For example, ~7,000 single nucleotide polymorphisms (SNPs), 597 in coding regions, and 432 non-synonymous, distinguish ramets of a large Finnish clone of the seagrass *Zostera marina* (Yu et al., 2020). To set this in perspective, 139,321 biallelic SNPs were reported in coding regions among four populations of the same species (Jueterbock et al., 2016). The degree to which epigenetic variation can contribute to phenotypic heterogeneity in ecologically relevant traits, independently from the underlying genetic variation, is a key question in assessing its contribution to stress tolerance, and long-term survival of clonal organisms.

The definition of epigenetics is currently heavily debated (Ptashne, 2007; Greally, 2018). Here, “epigenetics” implies molecular variations that do not alter the DNA sequence but have the potential to change gene expression, and include non-coding RNAs (ncRNAs), histone modifications, and DNA methylation (Bossdorf et al., 2008). DNA methylation is, from an evolutionary perspective, the most relevant epigenetic mechanism because it can be independent from genetic variation (Bossdorf et al., 2008; Schmitz et al., 2013; Kilvitis et al., 2014), and transgenerationally stable (Boyko et al., 2010; Verhoeven et al., 2010; Ou et al., 2012; Bilichak et al., 2015; Williams and Gehring, 2017). DNA methylation involves the addition of a methyl-group to the C5 position of a cytosine in DNA sequence motifs (CG, CHG, and CHH in plants, where H stands for A, C, or T) (Kilvitis et al., 2014). Depending on sequence context, methylation can be associated with gene activation or silencing (Bossdorf et al., 2008; Niederhuth and Schmitz, 2017). While CG methylation in gene bodies often correlates with increased gene expression, methylation in promoters and repeat regions, such as transposable elements (TEs), silences expression (Feng et al., 2010; Seymour et al., 2014; Dubin et al., 2015; Bewick and Schmitz, 2017; Zhang et al., 2018).

The methylome, or set of DNA methylation modifications in an organism’s genome, can change spontaneously at a rate of 2.5 × 10^−4^ to 6.3 × 10^−4^ methylation polymorphisms per CG site per generation, which is about 7 × 10^4^ higher than the genetic mutation rate of base substitutions per site per generation (Schmitz et al., 2011; van der Graaf et al., 2015). That methylome variation can enhance productivity, and pathogen resistance, has been shown in *Arabidopsis thaliana* plant populations (Latzel et al., 2013). Moreover, methylation variation explained the rapid invasive success of the Japanese knotweed (*Fallopia japonica*) by facilitating differentiation in response to new habitats despite decreased genetic variation (Richards et al., 2012). This suggests that methylation variation complements genetic variation as a source of phenotypic variation in plant populations deprived of genotypic diversity (Gao et al., 2010; Richards et al., 2012; Verhoeven and van Gurp, 2012; Latzel et al., 2013; Zhang et al., 2013; Vanden Broeck et al., 2018).

Unlike genetic variants, methylation variants can also switch state directly in response to the environment (Dowen et al., 2012) and, if stable enough, establish a molecular memory that can be involved in stress priming. Under clonal growth, DNA methylation patterns are expected to be more stably inherited than under sexual reproduction (Verhoeven and Preite, 2014), because clonal growth circumvents epigenetic reprogramming during gameto- and embryogenesis. Although stable transmission across asexual generations has been shown for environment-specific phenotypes and DNA methylation patterns in clonal plants (Verhoeven et al., 2010; Verhoeven and van Gurp, 2012; Vanden Broeck et al., 2018; Verhoeven et al., 2018), a link between methylation variation and fitness-related traits has yet only been demonstrated in non-clonal plants (Latzel et al., 2013). Thus, while epigenetic mechanisms have been suggested to contribute to clonal plant success (Douhovnikoff and Dodd, 2014; Verhoeven and Preite, 2014; Dodd and Douhovnikoff, 2016; Latzel et al., 2016), empirical evidence is virtually lacking.

Clonal propagation is especially well-developed in aquatic plants (Barrett, 2015). Seagrasses, the only plants to inhabit the marine world, form the foundational basis of some of the most productive and highly diverse coastal marine ecosystems on the planet, and are essential for the health and abundance of economically exploited marine species (Costanza et al., 1997; Larkum et al., 2006; Orth et al., 2006). Ecosystem services are worth more than € 25,000 ha^−1^ year^−1^ (Costanza et al., 2014), including nursery grounds, habitat and food for fish and invertebrates, protection of the coastline from erosion, carbon sequestration of up to 186 g C m^2^ yr^−1^ (Fourqurean et al., 2012; Duarte et al., 2013), and nutrient fixation (Orth et al., 2006; Procaccini et al., 2007).

Over the last decades, losses of seagrass ecosystems have been documented worldwide due to increasing anthropogenic stressors such as invasive species, sediment and nutrient runoff, dredging, aquaculture, rising sea levels, and global warming (Orth et al., 2006; Chefaoui et al., 2018). Losses are expected to accelerate under projected global temperature increase, as ocean warming is considered the most severe threat among climate change factors (Repolho et al., 2017; Duarte et al., 2018) and seagrass meadows have tracked temperature changes in the past (Olsen et al., 2004). How accurate these shifts and losses can be predicted depends on our knowledge of drivers of adaptive potential, including epigenetic diversity (Duarte et al., 2018).

In this study, we characterize the functional relevance of epigenetic variation for heat stress resilience in the seagrass *Zostera marina*. *Z*. *marina* is the most widely distributed seagrass in the northern hemisphere, inhabiting highly contrasting habitats from sub-arctic to sub-tropical waters (Green and Short, 2004; Olsen et al., 2004; Boström et al., 2014). Few plants display such dramatic range in clonal diversity as *Z*. *marina* (Reusch et al., 2000; Olsen et al., 2004). Its clonal architecture varies from genetically diverse meadows with high levels of sexual reproduction, to meadows composed of a single large clone due to exclusive vegetative reproduction (Reusch et al., 1999; Olsen et al., 2004; Becheler et al., 2010; Reusch and Boström, 2011). Clonality of *Z*. *marina* peaks in the Baltic Sea, where large clones were estimated > 1,000 years old (Reusch et al., 1999). These meadows display remarkable phenotypic plasticity and persistence in time under drastic changes in ice cover, salinity, and temperature (Reusch et al., 1999), and under perturbations that represent environmental stress predicted elsewhere for the future (e.g. high temperatures) (Reusch et al., 2018). Moreover, extension of a single seagrass clone in space over ca. 160 × 40 m, and a water depth ranging from 1.5 to 4.5 m with an environmental gradient in light, sedimentation, wind exposure, and ice scour, strongly suggests niche differentiation among ramets of a single clone (Reusch et al., 1999). Thus, clonal meadows (such predominated by a single genet) confound experimental results showing a positive effect of genotypic (i. e. clonal) richness on the productivity and stress resilience of *Z*. *marina* (Hughes and Stachowicz, 2004; Reusch et al., 2005; Reusch and Hughes, 2006; Ehlers et al., 2008; Hughes et al., 2008). In other words, if stress resilience and tolerance would rely strongly on genotypic diversity, clones would not be able to disperse widely in space or survive for such long time periods.

The resilience, longevity, and adaptive potential of clonal seagrass meadows remain unknown without a fundamental analysis of their epigenetic variation and its ecological relevance. Therefore, this study tests the hypothesis that variation in DNA methylation can promote functional phenotypic diversity and, thus, may explain how clonal seagrass meadows can persist over millennia.

Specific objectives were to: 1) Characterize DNA methylation variation in an ancient clonal meadow of *Z*. *marina* (>1,000-years-old); and 2) Identify the functional role of this variation in photosynthetic performance—specifically how photosynthetic performance under benign and stressful temperatures is linked to DNA methylation variation independently from underlying somatic mutations.

## Materials and Methods

### Sampling and Cultivation

**Figure 1:**
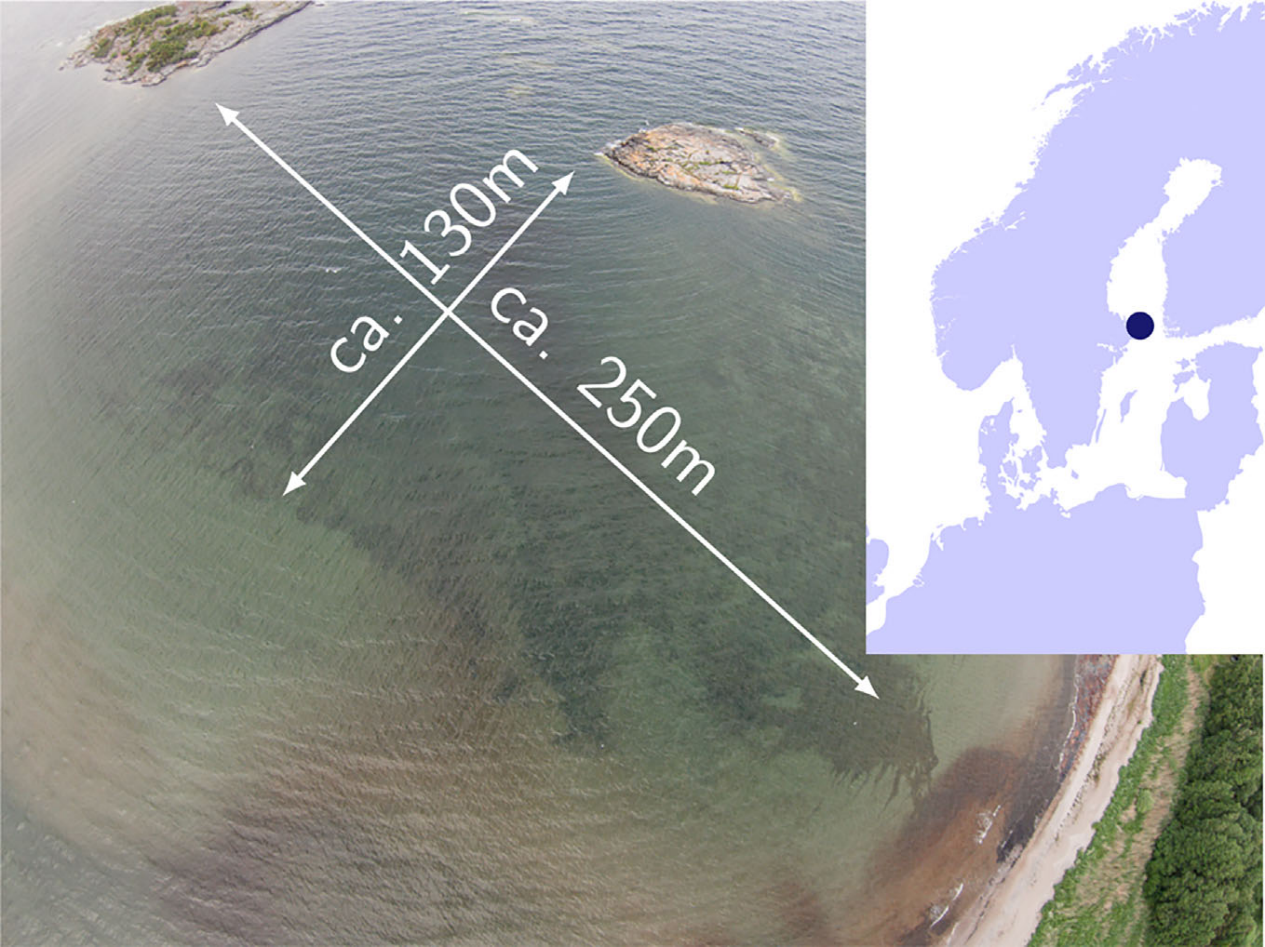
Baltic Sea sampling site of a > 1,000 years old clonal meadow where 42 *Zostera marina* shoots were collected along a 250 m transect.

In June 2015, we sampled seagrass shoots from a clonal meadow (Reusch et al., 1999) in the Baltic Sea (Åland Islands, 60°09′50.4′′N, 19°31′48.1′′E) (**Figure 1**) by collecting two to three shoots attached to the same rhizome (ramets) every 3 meters along a 250 m transect (**Supplementary Table S1**). Along the transect running perpendicular to the shore, water depth increased from 0.5 to 3 m. Shoots were transported in seawater-filled cooling boxes to the field station at Nord University, Norway. The most mature leaf of 42 shoots (field transect samples), randomly chosen from the two to three connected ramets, was flash frozen in liquid nitrogen for subsequent DNA extraction (**Supplementary Table S1**). Thus, recorded DNA methylation patterns are expected to reflect the average state of methylation of a mature leaf, along which global DNA methylation has been shown to vary with tissue age in the seagrass Posidonia oceanica (Ruocco et al., 2019b).

Ten ramets of the same genet (**Supplementary Table S1**), sampled at the same time and from the same transect as the field samples, were individually replanted in plastic containers (10 × 15 cm) filled with sand from the sampling site, placed in a 1,280-L aquarium at 15°C (corresponding to field temperature), and illuminated with a 16:8 h light–dark cycle, under light-saturating conditions (200-220 µmol m^−2^ s^−1,^OSRAM Fluora, 150 W) (Dennison and Alberte, 1982; Alcoverro et al., 1999). Seawater at 5.5 PSU, corresponding to the salinity at the collecting site, was obtained by mixing freshwater and natural filtered seawater at 32 PSU. The water, filled to a 40-cm level, was kept in constant motion with airstones. Once a week, 50% of the water was renewed after removing epiphytic algae.

### Heat Stress Experiments

After two weeks of acclimation, the 10 clonal ramets were exposed to heat stress (**Supplementary Table S1**) in a climate chamber (Fitotron, weisstechnik), where they were distributed in three aquaria (60 L, 3–4 ramets in each aquarium) filled with aerated brackish water (5.5 PSU) to 30 cm, of which 50% was weekly renewed. Light was kept at 240 µmol m^−2^ s^−1^ and a 16:8-h light:dark cycle. The temperature in the climate chambers was increased with a daily increment of 3°C from 15°C to 27°C, which can be lethal for *Z*. *marina* (Greve et al., 2003) and exceeds maxima of sea surface temperatures recorded in the Baltic Sea by ca. 2°C (Reusch et al., 2005), but is likely to be reached in the Baltic Sea during future summer heat waves. After three weeks at 27°C, the temperature was decreased by a daily increment of 3°C from 27°C to 15°C, after which all shoots were returned to the 1,280 L aquarium at 15°C for a recovery period of 5.5 weeks where they were exposed to the same conditions as under acclimation.

At three time points (control, stress, recovery), one entire mature (outer-most) leaf of each shoot was excised and flash-frozen in liquid nitrogen. Thus, the methylation pattern of each sample represents the average methylation state across young and mature tissues, which have been shown to vary in their epigenetic heat-stress response in the seagrass *Posidonia oceanica* (Ruocco et al., 2019a). Control samples were collected at 15°C on the day before the temperature was increased. Stressed samples were collected at 27°C on the day before the temperature was decreased. Recovery samples were collected after the 5.5-week recovery period at 15°C.

### Photosynthetic Performance

At control, stress, and recovery time points, we measured for each shoot (two measurements for each of the two inner leaves) the increase in chlorophyll *a* fluorescence upon illumination after a 10 min dark period (OJIP curve) (Bussotti et al., 2010), with a PAM-Fluorometer (FluorPen FP100, Photon Systems Instruments) using a saturating pulse of 75% light intensity at 455 nm. From the measurements, we extracted the photosynthetic performance index PiABS (Strasser et al., 2000), reflecting the functionality of photosystem II (PSII) and photosynthetic performance in general (Živčák et al., 2008; Bussotti et al., 2010; Stefanov et al., 2011). PiABS combines three parameters: 1) the density of reaction centers, 2) the efficiency of electron transport beyond Q_A_ at the onset of illumination, and 3) the probability for an absorbed photon to reach the reaction center in PS II. PiABS is calculated as: PiABS = ((1 − F_0_/F_M_)/(M_0_/V_J_)) × ((F_M_ − F_0_)/F_0_) × ((1 − V_J_)/V_J_). Here, F_0_ is the minimum fluorescence intensity in a dark adapted leaf when all reaction centers are open (all quinone acceptors are oxidized), F_J_ is the fluorescence intensity at 2 ms illumination, F_M_ is the maximum fluorescence intensity when all reaction centers are closed (all quinone acceptors are reduced), V_J_ is the relative variable fluorescence at 2 ms calculated as V_J_ = (F_J_F_0_)/(F_M_F_0_), and M_0_ reflects the initial slope of fluorescence kinetics, and is calculated as M_0_ = 4 × (F_300__μs_F_0_)/(F_M_F_0_) (Živčák et al., 2008). We tested for correlation of PiABS values (mean values per shoot) between time points using a two-sided Pearson’s product moment correlation test in R v3.4.4 (R Core Team, 2019).

### DNA Extraction

All flash-frozen tissue was freeze-dried for one day, then stored at −80°C until DNA extraction. DNA was extracted with the HP Plant DNA mini kit (Omega Bio-Tek, protocol version May 2013) from ≥ 5 mg of lyophilized leaf tissue after grinding with a mixer-mill (Retsch MM 400) in 2 mL Eppendorf tubes with tungsten beads supplied (60 s at 30 Hz). We added 10 µl beta-mercaptoethanol at step 2, and equilibrated the columns at step 8 of the standard protocol. The extracted DNA was eluted in 2 × 100 µl EB buffer (Qiagen), cleaned, and concentrated with the Clean and Concentrator-5 kit (Zymo Research, protocol v1.2.1) using 15,000 g for all centrifugation steps, and finally eluted in 2 × 30 µl EB Buffer (Qiagen) at 60°C.

### Clonality

In order to identify ramets belonging to the same genet, we genotyped each shoot for seven microsatellite loci (ZosmarGA2, -GA3, -GA6, -CT3, -CT12, -CT19, -CT20) (Reusch and Boström, 2011). PCR was performed using a Veriti 96-Well Thermal Cycler (Applied Biosystems, Life Technologies) in two 10-µl multiplex reactions containing 2 µl cleaned genomic DNA, and 1× AccuStart II PCR ToughMix (Quanta bio). One multiplex reaction contained forward and reverse primers GA2, GA3, and GA6 at 0.5 µM each, and ran at 94°C for 4 min, followed by 30 cycles of 94°C for 60 s, 55°C for 90 s, and 72°C for 90 s, and a final extension at 72°C for 10 min. The other multiplex reaction contained forward and reverse primers CT3 (0.5 µM), CT12 (0.3 µM), CT19 (0.3 µM), and CT20 (0.3 µM), and ran at 94°C for 3 min, followed by 35 cycles of 94°C for 60 s, 57°C for 60 s, and 72°C for 60 s, and a final extension at 72°C for 10 min.

DNA fragment lengths were determined on an ABI 3500xl Genetic Analyzer from 1 µl of 1:99 diluted PCR products mixed with 8.9 µl of HiDi Formamide (Life Technologies), and 0.1 µl of Gene Scan 500 LIZ Size Standard (Life Technologies) after 5 min denaturation at 95°C. Alleles were called with the GeneMapper v4.1 Software (Applied Biosystems, Thermo Fisher Scientific). The shoots were assigned to multi-locus genotypes using the R package “RClone” (Bailleul et al., 2016).

### Whole Genome Sequencing, SNP Detection, and Genetic Distance in Experimental Samples

In order to detect SNPs resulting from somatic mutations in the 10 heat-stressed ramets, genomic DNA libraries were prepared according to the TruSeq DNA PCR-Free (Illumina) protocol, and sequenced on one Illumina HiSeq 3/4000 lane (2 × 150 bp) at the Norwegian Sequencing Centre (University of Oslo, Norway). Raw reads (25.7 million to 44.3 million per library, **Supplementary Table S2**, NCBI BioProject PRJNA575339) were quality-checked with FastQC v0.11.8^1^ to control for aberrant read base content, length distribution, duplication, and over-representation. We used TrimGalore! v0.6.0^2^ to remove adapter sequences with a stringency of 3 bp overlap, and low-quality bases with a Phred score Q < 20 (99% base call accuracy). The high-quality reads (25.5 to 44.0 million per library, **Supplementary Table S2**) were mapped to the *Z*. *marina* genome v2.1 (Olsen et al., 2016) with BWA v0.7.17 (Li and Durbin, 2009). Read duplicates were removed with MarkDuplicatesSpark within GATK v4.1.4.1 (Auwera et al., 2013). SNPs were called with HaplotypeCaller, followed by CombineGVCFs and GenotypeGVCFs within GATK v4.1.4.1 (Auwera et al., 2013).

Before filtering SNPs, we excluded indels, non-variant sites, and alternate alleles not present in any genotypes from the vcf file with SelectVariants within GATK v4.1.4.1 (Auwera et al., 2013). This set of 759,407 raw SNPs was reduced to 105,443 SNPs after hard-filtering with vcffilter from vcflib (Garrison, 2020) with thresholds that were based on density plots drawn with ggplot2 (Wickham, 2016): QualByDepth (QD < 15.0), FisherStrand (FS >12.0), RMSMappingQuality (MQ < 38), MappingQualityRankSumTest (MQRankSum < −1.5), ReadPositionRankSumTest (ReadPosRankSumand < −4.0), and Depth (DP > 4000.0, in order to remove SNPs potentially caused by genome duplication). Subsequently, we used VCFtools v0.1.15 to remove genotypes with genotype quality < 30 (–minGQ 30) or depth < 20 (–minDP 20), and to remove SNPs with more than 2 alleles (–min-alleles 2 –max-alleles 2), with a minor allele frequency of 0.01 (–maf 0.01), and with any missing genotype (–max-missing-count 0). From the remaining 15,508 high-quality SNPs (**Supplementary File S1**) we excluded all that shared the same genotypes among all 10 heat-stressed shoots, as these reflected genetic differences only to the reference genome. The remaining 1,079 SNPs (**Supplementary File S2**) were used to estimate Euclidean genetic distances among the 10 shoots using the R package vcfR v1.9.0 (Knaus and Grünwald, 2017) and the *dist* function of the R package “stats” v3.6.9 (R Core Team, 2019). We tested for correlation between genetic and physical distance among the 10 heat-stressed shoots with Mantel tests in the R package “vegan” v1.4-2 (Oksanen et al., 2019), using 1,000 permutations, and the Pearson’s product moment correlation method.

### Methylome Characterization

Sequencing libraries were prepared according to the MethylRAD protocol (Wang et al., 2015) with few adjustments. MethylRAD is a genome-reduction method based on the methylation-dependent restriction enzyme FspEI that targets fully methylated CCGG and CCWGG motifs, thus capturing methylation in CG and CHG sequence contexts. MethylRAD has the potential to reveal genome-wide DNA methylation patterns that are consistent with those generated from Whole Genome Bisulfite Sequencing (Wang et al., 2015). First, sense and anti-sense oligos of adapters A1 and A2 (**Supplementary File S3**) were annealed in 10 µl containing 10 µM of each oligo (Eurofins), 10 mM Tris HCl (Thermo Fisher), 50 mM NaCl (Thermo Fisher), and 1 mM EDTA (Thermo Fisher). Library preparation began with digestion of 100 ng cleaned genomic DNA at 37°C for 4 h in 15 µl containing 4 U FspEI (NEB), 1× CutSmart Buffer (NEB), and 30× Enzyme Activator Solution (NEB). Digestion was verified on a TapeStation 2200 with a D1000 ScreenTape. Second, adapters were ligated to the digested fragments over night at 4°C in 26 µl containing 13 µl digestion solution, 0.1 µM each of two annealed adapters, 1× T4 ligase buffer (NEB), 1.5 µM ATP (NEB) and 1040 U of T4 DNA ligase (NEB). Ligation products were amplified in 20-µl reactions containing 7 µl ligated DNA, 0.05 µM of each primer (P1 and P2, **Supplementary File S3**), 0.3 mM dNTP, 1× Phusion HF buffer (NEB) and 0.4 U Phusion high-fidelity DNA polymerase (NEB). PCR was conducted using a Veriti 96-Well Thermal Cycler (Applied Biosystems, Life Technologies) with 16 cycles of 98°C for 5 s, 60°C for 20 s, 72°C for 10 s, and a final extension of 5 min at 72°C. The target band (approx. 100 bp) was extracted from a 2% E-Gel (Thermo Fisher). For multiplex sequencing, shoot barcodes were introduced by means of PCR. Each 20 µl PCR reaction contained 12 µl of gel-extracted PCR product, 0.2 µM of each primer (P3 and index primer, **Supplementary File S3**), 0.3 mM dNTP, 1× Phusion HF buffer (NEB) and 0.4 U Phusion high-fidelity DNA polymerase (NEB). PCR was conducted with the same PCR cycling program outlined above. PCR products were purified using AMPURE XP beads (Beckman Coulter) using a 1.8:1 volume ratio of beads to product, and a final elution in 22 µl EB buffer (Qiagen). The purified fragments were sequenced on an Illumina NextSeq 500 (1 × 75 bp) using three high-output flow-cells: The 10 experimental samples were sequenced on a single flow-cell, while the field transect samples were split over two other flow-cells (detailed in **Supplementary Table S3**).

The sequenced reads were quality-trimmed with TrimGalore! v0.4.1^3^ by removing the adapter sequences with a stringency of 3 bp overlap, low-quality bases with a Phred score Q < 20, and the terminal 2 bp from both ends in order to eliminate artifacts that might have arisen at the ligation position. Quality was checked with FastQC v0.11.8^4^ to control for aberrant read base content, length distribution, duplication and over-representation.

The high-quality reads were mapped with SOAP v1.11 (Li et al., 2008) to 628,255 *in silico* predicted MethylRAD tags that were extracted from the *Z*. *marina* genome v2.1 from ORCAE (Sterck et al., 2012) with the custom python script *InSilicoTypeIIbDigestion.py^5^*. For mapping, we allowed for two mismatches, filtered reads with >1 N, and used a seed size of 8 bp. Based on the uniquely mapped reads, we counted the coverage of each methylated site for each shoot using htseq-count (v0.7.2). Methylation calls were retained only for sites with ≥ 2× coverage, which reduced the false-positive rate from 1.10% to 0.23% for CG sites and from 2.50% to 0.89% for CHG sites. False-positive rates were estimated as the percentage of methylation sites supported by at least two reads in the generally unmethylated chloroplast genome. For each shoot, raw counts were normalized to reads-per-million by dividing reads per site through the total number of reads per shoot library, times one million.

The methylation sites were annotated with the v2.1 gff3 file from ORCAE (Sterck et al., 2012), and separated into genes, intergenic regions, and TEs. TEs were located in both genes and intergenic regions, and contained repeats classified by RepeatModeler^6^ as rnd-1/2/3/4/5 families, referring to the series of processing rounds of the *de novo* TE family identification program. The methylation sites were further separated into such containing CG and CHG recognition sites. Sequence contexts for all 628,255 *in silico* predicted MethylRAD tags are listed in **Supplementary Table S4**.

### Methylation Variation Between the Field Transect Shoots

In order to describe the level of intra- and inter-clonal epigenetic distance among the 42 transect shoots (of which shoot 27 belonged to a different genet than the 41 other ramets, **Supplementary Table S1**), we calculated the Euclidean distance between shoots based on their coordinates in the 2-dimensional PCA (Principal Component Analysis) plot using the *dist* function of the R package “stats” v3.6.0 (R Core Team, 2019). PCA was done on reads-per-million for all shoots with the PCA function of the R package “FactoMineR” (Lê et al., 2008). We tested for correlations between epigenetic and physical distance among the 41 ramets with Mantel tests using the R package “vegan” v1.4-2 (Oksanen et al., 2019) with 1,000 permutations, and the Pearson’s product moment correlation method. *P*-values were adjusted according the Benjamini-Hochberg method (Benjamini and Hochberg, 1995).

### Correlation Between Methylome Variation and Photosynthetic Performance

For the 10 experimental samples, we tested for correlations between epigenetic distance and photosynthetic performance difference (PiABS values) among shoots, while controlling for genetic distance with Partial Mantel tests using the function *partial.mantel.test* of the R package “ncf” v1.2.9 (Lê et al., 2008). Only significant correlations (p<0.05, adjusted for multiple comparisons according to (Bjornstad, 2020) in R (R Core Team, 2019)) with coefficients *R* > 0.65, when controlled for genetic distance, were considered strong enough to be reckoned as biologically linked. The same analysis ran Mantel tests for correlation between genetic and epigenetic distance, and between genetic distance and photosynthetic performance difference.

### Methylome Shift Under Experimental Conditions

The methylome of the 10 heat-stressed shoots was characterized under control, stress, and recovery conditions (3 shoots died from the stress), and compared with the methylome of field transect samples with PCA on reads-per-million data using the PCA function of the R package “FactoMineR” (R Core Team, 2019). Epigenetic distances between the samples were estimated for each sequence context (gene, intergene, TE, each in CG and CHG regions, respectively) as the Euclidean distances in the 2-dimensional PCA plot using the *dist* function of the R package “stats” v3.6.9 (Lê et al., 2008).

### Differential Methylation Analyses

To estimate the number of sites that changed in methylation state from control to stress and recovery conditions, we applied differential methylation analyses using the R package “edgeR” 3.20.9 (R Core Team, 2019) within the “SARTools” pipeline v1.6.6 (Robinson et al., 2009). Read counts were normalized using a trimmed mean of M-values (TMM) between each pair of samples (Varet et al., 2016). All samples taken under control, stress, and recovery served as replicates for the three different conditions. Methylation levels were considered significantly increased (hyper-methylated) or decreased (hypo-methylated) when Benjamini-Hochberg adjusted *p*-values (Robinson and Oshlack, 2010) fell below 0.05.

**Figure 2:**
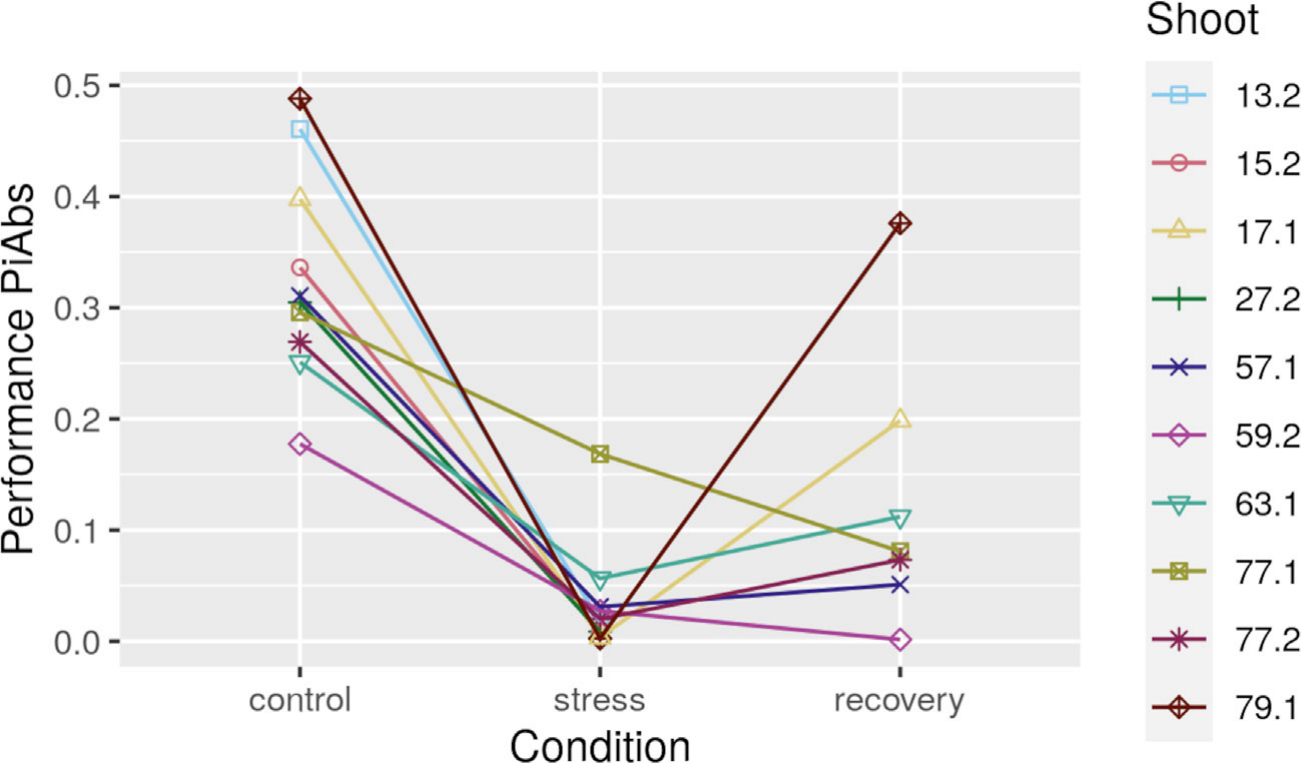
Photosynthetic performance (PiAbs, absolute values) for all ten heat-stressed *Zostera marina* shoots at control, stress, and after recovery. Shoots 13.2, 15.2, and 27.2 did not recover from the stress.

To identify how the methylome differed between samples of high and low photosynthetic performance, we used differential methylation analyses using the R package “edgeR” 3.20.9 (Benjamini and Hochberg, 1995) within the “SARTools” pipeline v1.6.6 (Robinson et al., 2009). Read counts were normalized using a trimmed mean of M-values (TMM) between each pair of samples (Varet et al., 2016). Differential methylation analysis was done only for conditions and sequence contexts where a positive correlation was found between photosynthetic performance differences and epigenetic distances. For control conditions, we compared methylation levels in genes (CG regions) between the two samples of highest photosynthetic performance (79.1, and 13.2) and the two samples of lowest photosynthetic performance (63.1 and 59.2, **Figure 2**). For recovery conditions, we compared methylation levels in intergenic TEs (CHG regions) between the two samples of highest photosynthetic performance (79.1 and 17.1) and the two samples of lowest photosynthetic performance (57.1 and 59.2). Methylation levels were considered significantly different between shoots of high and low photosynthetic performance, when Benjamini-Hochberg adjusted *p*-values (Robinson and Oshlack, 2010) fell below 0.05.

For differentially methylated sites within gene bodies, we tested with Fisher’s exact tests for enrichment of gene ontology terms of biological processes with the R package “topgo” (Benjamini and Hochberg, 1995) using Fisher’s exacts tests. GO terms were obtained from the v2.1 *Zostera* genome annotation from the ORCAE database (Alexa and Rahnenführer, 2010). To reduce redundancy in the significantly enriched GO terms (*p*-values < 0.05), we calculated “sim rel” scores (Sterck et al., 2012) (Allowed similarity=0.5), based on the *A*. *thaliana* GO-term database, using the REVIGO web server (Schlicker et al., 2006).

## Results

### Methylome Characterization and Variation Among the Clonal Transect Shoots

**Figure 3:**
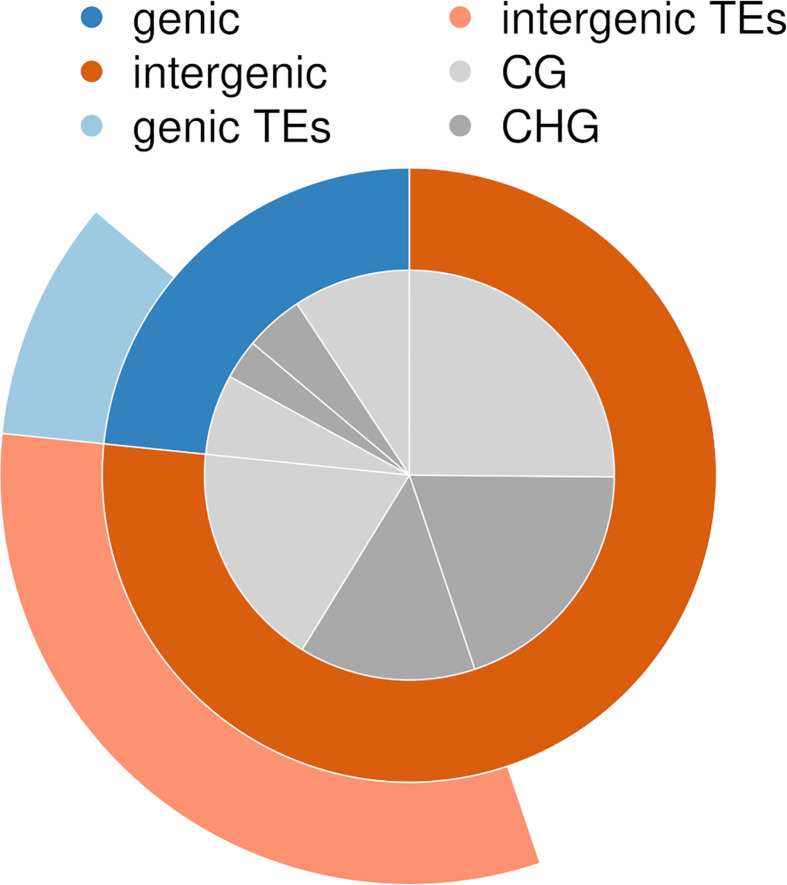
Proportion of methylated sites detected in genes, intergenic regions, and transposable elements (TEs), separated by CG and CHG sequence contexts.

On average, 74 million high-quality reads were obtained per sequencing library (*Methylome Characterization* section, ranging from 0.7 to 151 million (**Supplementary Table S3**). DNA raw reads are accessible from NCBI under BioProject number PRJNA575339. On average, 35% of the high-quality reads mapped to the *in silico* digested *Z*. *marina* genome, and 11% mapped uniquely (annotated reads-per-million in **Supplementary File S4**). In total, 144,420 sites were methylated (covered by at least two reads) across all transect shoots. Across all transect shoots, 84,640 methylated CG sites and 59,780 methylated CHG sites were detected, which represents 59% and 41% of all methylated sites, respectively (**Supplementary Table S5**). About 23% of all methylated sites fell in gene bodies (of which 41% in TEs), and 77% in intergenic regions (of which 42% in TEs) (**Figure 3**). In gene bodies, 67% of the methylated sites fell in CG regions, in intergenic regions only 56%.

**Figure 4:**
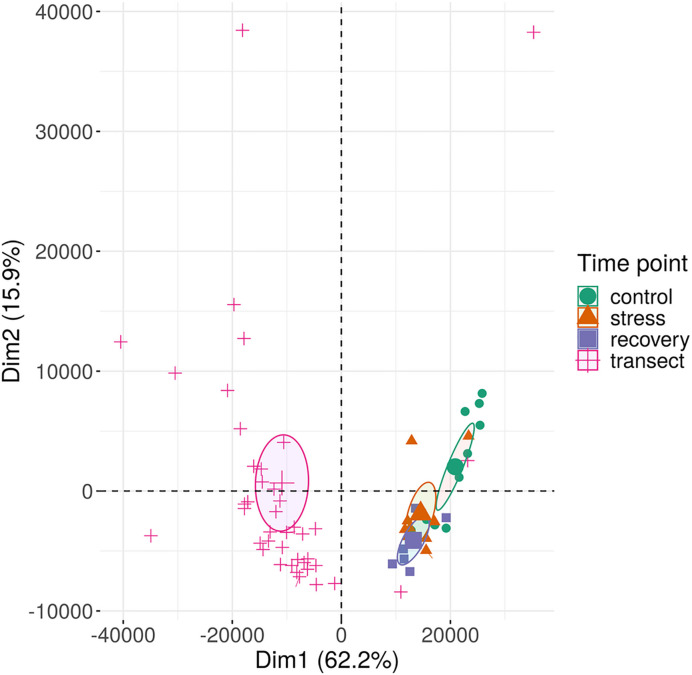
Methylation variation of field transect samples and of experimental samples of *Zostera marina* along the first two principal components. The samples (small symbols) are plotted along the first two principal components (Dim) based on methylation profiles across all sequence contexts. Circles represent 95% confidence intervals around group means (large symbols). Bracketed numbers represent the percentage of explained variation.

**Figure 5:**
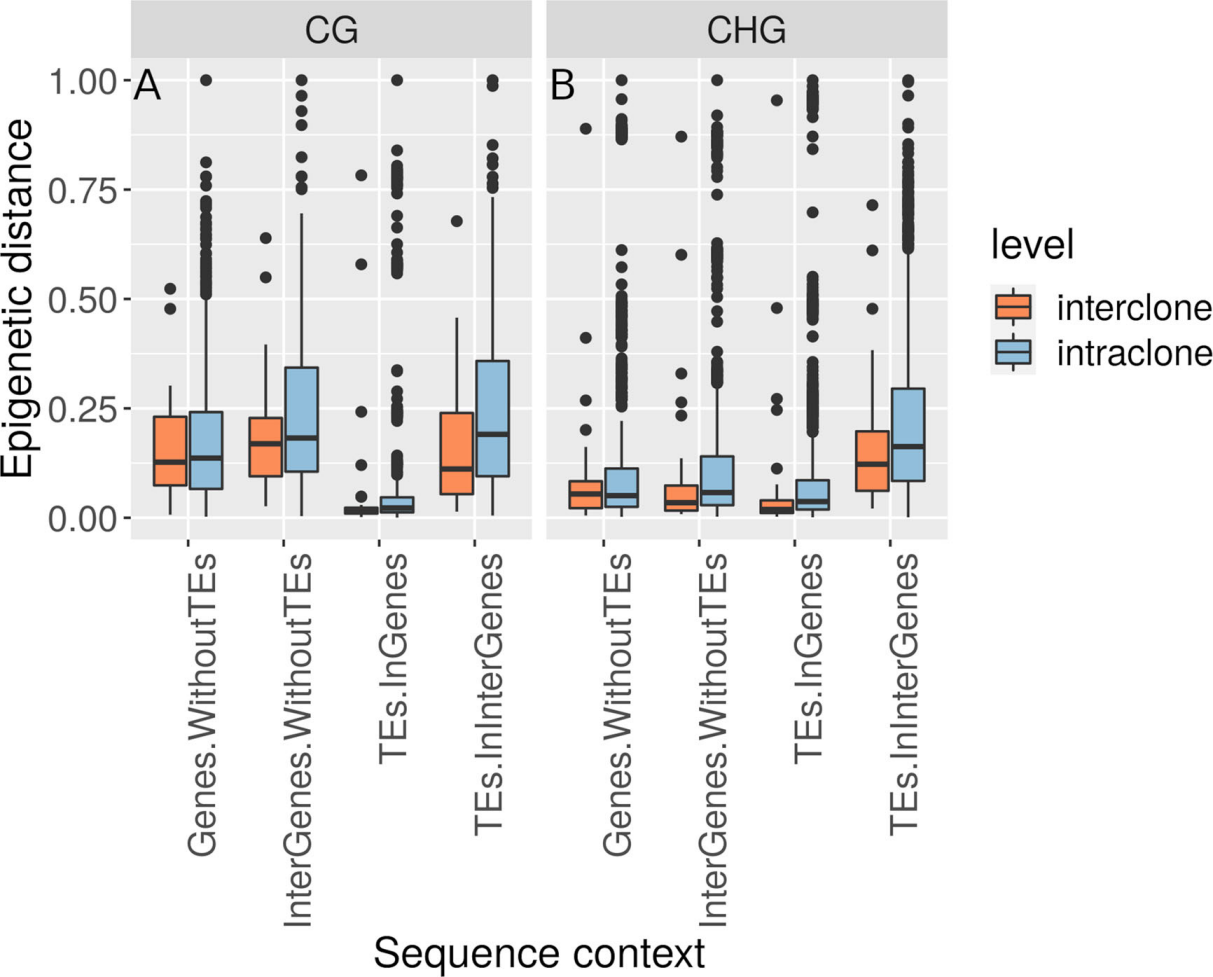
Boxplot of relative epigenetic distances (distances divided by the maximum distance for each sequence context) showing the median, upper and lower quartiles (25^th^ and 75^th^ percentiles, box margins), minimum and maximum values falling within 1.5 X the interquartile range above and below the box (ends of the vertical lines), and all outlying points individually. Epigenetic distances in **(A)** CG and **(B)** CHG regions were comparable within ramets of the same genet (intraclone) and between ramets of different genets (interclone) of *Zostera marina* across all sequence contexts.

Based on their multi-locus microsatellite genotype, 41 of 42 shoots sampled along the transect (except sample 27 in **Supplementary Table S1**) belonged to the same genet (*Clonality* section; **Supplementary Table S6**). Methylome variation between these 41 clonal ramets (*Methylation Variation between the Field Transect Shoots*) exceeded the methylome shift over the course of the heat-stress experiment (*Methylome Shift Under Experimental Conditions* section; **Figure 4**). Epigenetic distances between the 41 ramets of one genet and the single ramet of the other genet was not higher than among the 41 clonal ramets (*Methylation Variation between the Field Transect Shoots* section; **Figures 5A, B**, **Supplementary Table S5**). Epigenetic distances were generally lower in CHG than in CG sequence contexts, and lowest in TEs in gene regions (**Figures 5A, B**). Epigenetic distances were not significantly correlated with physical distances in any sequence context (*Methylation Variation between the Field Transect Shoots* section; **Supplementary Table S7**).

### Genetic Variation Among Heat-Stressed Shoots

Based on whole genome sequences of the 10 heat-stressed shoots, we identified 15,508 high-quality SNPs (*Whole Genome Sequencing, SNP Detection, and Genetic Distance in Experimental Samples* section; **Supplementary File S1**). That all 10 shoots shared the same heterozygous state in 14,429 (93%) of all SNPs, and shared the same multi-locus microsatellite genotype (*Clonality* section; **Supplementary Table S6**), suggests that they were clones having originated from one ancestral zygote (**Supplementary Figure S1**) (Supek et al., 2011).

Despite being clonal, the 10 shoots differed in 1,079 SNPs resulting from somatic mutations (*Whole Genome Sequencing, SNP Detection, and Genetic Distance in Experimental Samples* section). Based on these SNPs, Euclidean genetic distances ranged from 9 to 35 (frequency distribution in **Supplementary Figure S2**), and were not significantly correlated (*Whole Genome Sequencing, SNP Detection, and Genetic Distance in Experimental Samples* section) with physical distances between shoots (*R*=0.18, *p*=0.12).

### Link Between Epigenetic Distance and Photosynthetic Performance Changes Under Heat Stress

**Figure 6:**
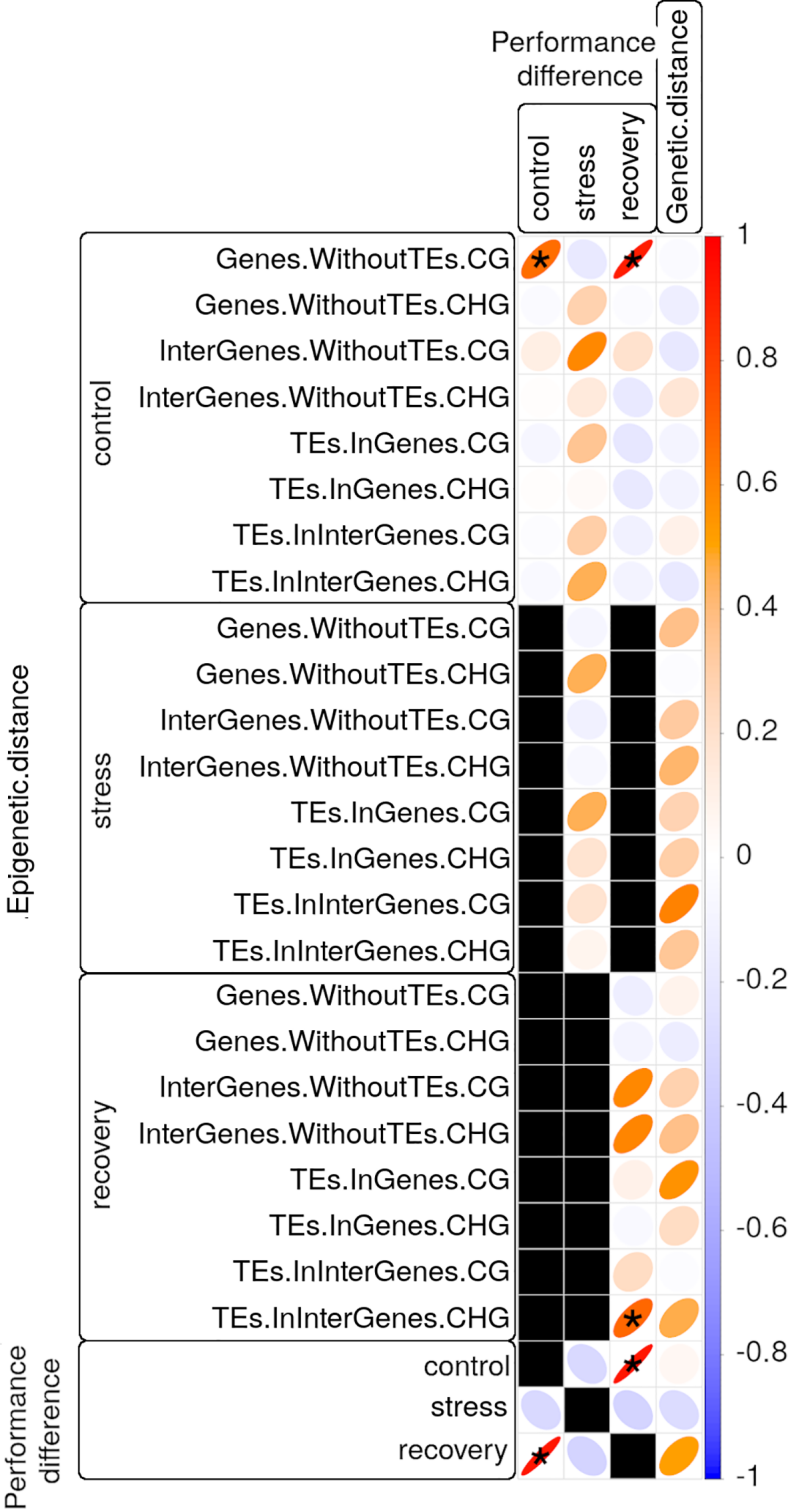
Correlation matrix of photosynthetic performance (PiABS) differences (first three columns) or of genetic distance (last column) among clonal *Zostera marina* shoots with epigenetic distances (first 24 rows) or with photosynthetic performance differences at control, stress, or recovery conditions (last three rows). All correlations between photosynthetic performance differences and epigenetic distances were controlled for genetic distances. Black squares represent untested correlations that were considered biologically not meaningful. Pearson product-moment correlation coefficients R are encoded by the color gradient explained in the bar on the right end, and by the shape of the ellipses. Narrow ellipses represent stronger correlations as compared with wide ones. Asterisks highlight strong (*R*>0.65) and significant (adjusted *p*<0.05) correlations. CG and CHG: sequence contexts of the methylated cytosine; TE: Transposable element.

Photosynthetic performance (PiABS, *Photosynthetic Performance* section) declined in all 10 shoots under heat stress (**Figure 2**, **Supplementary Table S8**, raw data in **Supplementary Table S9**). Seven of the 10 shoots recovered from the heat stress, yet photosynthetic performances did not reach pre-stress levels. Photosynthetic performances under control and recovery conditions were positively correlated (adjusted *p*<0.05, *R*=0.93, **Figure 6**, **Supplementary Table S10**).

Genetic distances correlated moderately (0.4<*R*<0.6, adjusted *p*<0.05) with photosynthetic performance differences in recovered samples, and with epigenetic distances (*Correlation between Methylome Variation and Photosynthetic Performance* section) in some sequence contexts of stressed and recovered samples (**Figure 6**, **Supplementary Table S10**). Nevertheless, epigenetic distances correlated strongly (*R*>0.65, adjusted *p*<0.05) with photosynthetic performance differences even after controlling for genetic distance based on 1,079 SNPs: 1) epigenetic distances among control samples in CG gene body regions correlated with photosynthetic performance differences prior to stress, and after recovery (stress resilience, **Figure 6**, **Supplementary Table S10**). However, some of the samples, including one that performed well before the stress (sample 13.2 in **Figure 6**), did not recover from the heat stress (*Photosynthetic Performance* section). Epigenetic distances among recovered samples in CHG regions of intergenic TEs correlated with photosynthetic performance differences after recovery (**Figure 6**, **Supplementary Table S10**).

### Methylome Changes in the Course of the Heat-Stress Experiment

**Figure 7:**
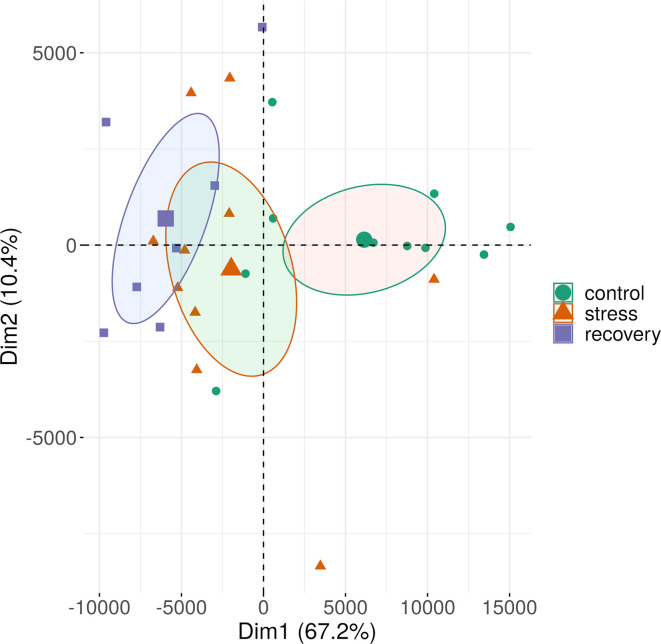
Methylation patterns of all sequence contexts in shoots of a *Zostera marina* over the course of the stress experiment. See Supplementary Figure S3 for methylation shifts separated by sequence context. The samples (small symbols) are plotted along the first two principal components (Dim) based on methylation profiles across all sequence contexts. Circles represent 95% confidence intervals around group means (large symbols). Bracketed numbers represent the percentage of explained variation.

**Figure 8:**
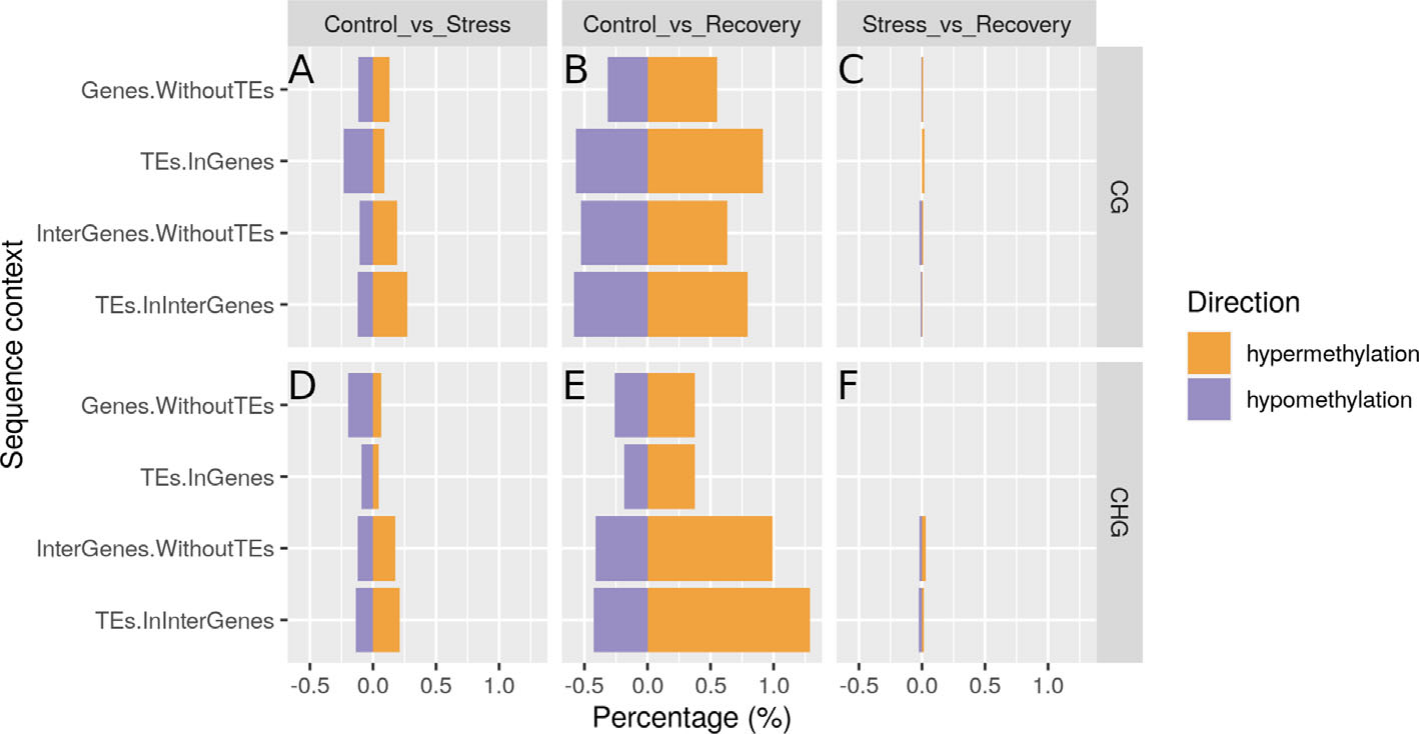
Hyper- and hypo-methylated sites in CG **(A–C)** and CHG **(D–F)** regions in stressed vs. control samples **(A, D)**, recovered vs. control samples **(B, E)**, and in recovered vs. stressed samples **(C, F)**. Hyper-methylation refers to higher methylation, hypo-methylation to lower methylation in samples taken during stress as compared with samples taken before stress **(A, D)** in samples taken after recovery as compared with samples taken before stress **(B, E)**, and in samples taken after recovery as compared with samples taken during stress **(C, F)**. TE, transposable element.

Over the course of the heat-stress experiment, methylation patterns in all sequence contexts changed and did not return to but instead diverged further from control (pre-stress) patterns during the recovery period (*Methylome Shift Under Experimental Conditions* section; **Figure 7** for all sequence contexts combined, **Supplementary Figure S3** for the different sequence contexts, **Supplementary File S5** listing annotated reads-per-million for each sample). More sites became hyper- (increased in methylation) than hypo-methylated (decreased in methylation) in the course of the experiment (*Differential Methylation Analyses* section 2.11; **Figures 8A, D**). Methylation levels differed significantly at 437 sites between control and stress conditions (257 hyper- and 180 hypo-methylated), at 1788 sites between control and recovery conditions (1141 hyper-, and 647 hypo-methylated), and only at 39 sites between stress and recovery conditions (18 hyper-, and 21 hypo-methylated, **Supplementary Table S11**). After recovery, CG methylation had changed more strongly in gene body regions, and CHG methylation in intergenic regions (**Figures 8B, E**).

After recovery, methylation increased (*Differential Methylation Analyses* section) in gene bodies with functions including DNA transcription and replication (GO:0006353, GO:0006261, GO:0006270); catabolism of misfolded proteins (GO:0006515), gamma-aminobutyric acid (GABA) (GO:0009450, GO:0009448), and neurotransmitter (GO:0042133, GO0042135, GO:0001505); as well as amino acid metabolism (GO:0009072, GO:0009073, GO:0009063) (**Supplementary Table S12**). DNA methylation had decreased in gene bodies with functions including transmembrane transport of cations, ions, and protons (GO:0006811, GO:0055085, GO:0006810, GO:0034220, GO:0006812, GO:0098655, GO:0098660, GO:0015672, GO:0099132, GO:0015991, GO:0098662, GO:0099131, GO:0015988, GO:1902600); transport of ammonium and phospholipids (GO:0072488, GO:0015696, GO:0015914, GO:0006869); localization of lipids and organelles (GO:0051179, GO:0051234, GO:0010876, GO:0051640); exocytosis (GO:0006887, GO:0006904); and secretion (GO:0032940) (**Supplementary Table S12**).

### Differential Methylation Between Shoots of High and Low Photosynthetic Performance

Recovered shoots of high and low photosynthetic performance presented too similar methylation patterns to allow for detection of many regions showing significant differentiation in methylation levels (*Differential Methylation Analyses* section). Only four CHG sites in intergenic TEs were hyper-methylated in recovery samples of high photosynthetic performance (**Supplementary Table S13**). In contrast, 90 CG gene body sites were hyper-methylated (1 hypo-methylated) in control samples of high photosynthetic performance (**Supplementary Table S13**). Enriched biological processes in the 90 hyper-methylated CG sites included “light harvesting in photosystem I” (GO:0009768, GO:0009765) and “protein folding” (GO:0006457, GO:0042026, GO:0006515) (**Supplementary Table S14**).

## Discussion

Plant genets persisting >1,000 years challenge the positive correlation between genetic variation retained through recombination, and long-term survival. Our study shows for the first time that ramets of the same seagrass genet display DNA methylation variation that is independent from underlying genetic variation, and associated with phenotypic variation in the fitness-related trait of photosynthetic performance under experimental conditions. To what degree this association could have been affected by transplantation-induced stress responses in the methylome remains unknown, given the strong methylome shift under acclimation to laboratory conditions. The lack of untreated control samples in the heat stress experiment did not allow us to distinguish methylome shifts induced by acclimation from such induced by heat stress. Nevertheless, our findings support the hypothesis (Yu et al., 2020) that methylation variation, *via* variation in gene regulation, compensates potential costs of clonal reproduction (Dodd and Douhovnikoff, 2016), and contributes to the long-term survival of clonal seagrass meadows by increasing variation in ecologically relevant traits that cannot be simply explained by the underlying somatic genetic variation.

### Methylome Variation of Functional Relevance

Methylome variation among ramets of the same genet resulted either from random epimutations or from microscale variations in the environment because its correlation with physical distance between shoots was weak. By sampling entire leafs composed of young and mature tissue, we aimed to standardize variation in tissue maturity among shoots, since tissue maturity can affect methylation patterns (Lynch et al., 1993; Lynch and Lande, 1998). None of the shoots had been reproductive at the time of sampling, so that variation in reproductive status is unlikely to explain the recorded methylome variation among shoots (Ruocco et al., 2019b).The change in depth of 3 meters and, thus, gradual changes in environmental factors along the sampled transect may not have been extreme enough to impose a result. The disagreement between methylome variation and transect position may also result from recent uprooting and re-settling of some shoots. This was suggested to explain disagreement between genetic similarity of clonal shoots and their transect position in another clonal meadow of *Z*. *marina* (Marín-Guirao et al., 2019), and would further explain the absence of correlation between genetic and physical distance in our heat-stressed ramets. In such case, the re-settled shoots would display a methylome shaped by distant environmental conditions.

Epigenetic distance was comparable at the intra- and inter-clonal level at all sequence contexts (**Figures 5A, B**). It is important to note that the inter-clonal comparisons relied on 41 shoots of one genet versus a single shoot of another genet. That we could not identify more genets in our samples supports the finding that this meadow is dominated by a single genotype (Reusch et al., 1999). It is unlikely that this genet could belong to the same genet as the other 41 shoots with a somatic mutation explaining its different microsatellite genotype at locus ZosmarCT19 (heterozygous with a 147bp fragment, and not homozygous for the 150bp fragment as the other shoots). This microsatellite locus had been developed as part of a set of seven less variable loci to discriminate genets, in contrast to other hypervariable microsatellite loci that allow to identify somatic mutations (Yu et al., 2020). However, the inter-clonal comparisons have to be taken with caution as it remains unknown how different the methylation profile of one or more additional genets from the same meadow could be.

The seagrass methylome is flexible and responds directly to environmental change, given its strong change from field transect samples to acclimated control samples within two weeks (**Figure 4**). Three factors may explain why the shift in methylation patterns appears stronger in response to acclimation to laboratory conditions than in response to heat stress (**Figure 4**). First, several environmental factors had likely changed from field to lab conditions, including light intensity, and nutrient composition of the water. The wider variation in methylation profiles among field samples than among lab samples may mirror microscale variations in the environment and appears to have converged to more similar methylation patterns under controlled and uniform laboratory conditions. Although the applied heat stress (27°C) was strong, only temperature had changed over the course of the experiment, potentially requiring less changes in methylation state as compared with lab acclimation. Second, field and acclimation epigenotypes differed already before the acclimation. Thus, the methylation changes from field to lab conditions can not only be ascribed to changes in environmental conditions but also to intrinsic differences between the epigenotypes. This applies to a lower extent to the experimental samples, because the same epigenotypes had been sampled throughout the experiment. Third, since we had not run technical replicates on the three sequencing flow-cells, part of the methylome shift under acclimation may have been due to flow-cell batch effects. All experimental samples had been run on a different flow cell than the field transect samples, which had been run on two other flow-cells (**Supplementary Table S3**). Although the 95% confidence intervals of methylation patterns of the field transect samples from different flow-cells did not overlap, the patterns were still more similar between all field samples than between field and experimental samples (**Supplementary Figure S4**). This does not alter the conclusion that there has been a shift in methylation patterns from field to lab conditions, because it is supported by two independent sequencing runs. However, this shift may be weaker than shown in **Figure 4**, due to sequencing batch effects.

Methylome variation in CG gene regions predicted photosynthetic performance (**Figure 6**) and, thus, is likely functionally relevant for this fitness-relevant trait. Methylation variation did not predict photosynthetic performance under acute heat-stress, given that the photosynthetic performance of the different epigenotypes converged to low and highly similar photosynthetic performance values under stress (**Figure 2**). Although difficult to prove (Reusch and Boström, 2011), the correlations between methylome and photosynthetic performance differences before and after the heat-stress may be causal, given that control samples of high photosynthetic performance showed hyper-methylated CG sites in gene bodies with relevant functions: “light harvesting in PSI” (GO:0009768), “protein folding” (GO:0006457), “protein refolding” (GO:0042026), and “misfolded or incompletely synthesized protein catabolic process” (GO:0006515) (**Supplementary Table S14**). While increased light harvesting can enhance photosynthetic performance, the functions related to protein folding can prime against heat stress when increased methylation is associated with expression of the underlying genes and, thus, with accumulation of protective molecules like heat-shock proteins. Heat shock proteins are involved in repair and prevention of heat-induced protein damage (Crisp et al., 2016), and that can facilitate a fast stress response. Indeed, while genes are generally repressed by methylated promoter regions (Feder and Hofmann, 2002), genes are often activated by CG gene body methylation (Lisch, 2009) that prevents aberrant expression from intragenic promoters (Zhang et al., 2006; Schmitz et al., 2013; Yang et al., 2014; Dubin et al., 2015; Bewick and Schmitz, 2017; Niederhuth and Schmitz, 2017). Accordingly, in the seagrass *Posidonia oceanica* hyper-methylation was associated with transcriptionally active leaf segments (Takuno and Gaut, 2012; Neri et al., 2017), and appears to be a common response to stress (Greco et al., 2012, 2013; Ruocco et al., 2019a). Thus, *via* association with gene expression, the observed methylome variation in control samples may putatively create ecologically relevant phenotype variation that predicts photosynthetic performance. However, it is important to note that the methylome variation in control samples does not reflect the methylome variation present in the field but may have already been shaped by transplantation-related stress, given the strong methylome shifts under acclimation to laboratory conditions. It is also important to note that the methylome of a high-performing shoot does not always protect from stress, given that one of the highest-performing samples (shoot 13.2 in **Figure 2**) did not recover from the heat stress.

Only four CHG sites in intergenic TEs were differentially methylated between the recovered samples of high and low photosynthetic performance (**Supplementary Table S13**). This appears to contradict the correlation between methylation patterns and photosynthetic performance. However, especially the samples of the high-performance group (79.1 and 17.1) showed a big difference in photosynthetic performance values between themselves (**Figure 2**) and, thus, were likely also too different in methylation patterns to allow for the detection of sites being differently methylated as compared with the samples of the low-performance group.

Although our study was based on clonal ramets that presented identical seven-locus microsatellite genotypes, the 10 heat-stressed ramets varied genetically at 1,079 SNPs generated by somatic mutations. Epigenetic differentiation between populations can be tightly linked to genetic differentiation (Ruocco et al., 2019b). Accordingly, trans-acting SNPs appeared to explain the correlation between gene body methylation and latitude in *Arabidopsis* (Herrera and Bazaga, 2010; Gáspár et al., 2019). In contrast, part of the correlation between DNA methylation with ecological factors in landscape epigenomic studies on non-model plant species could not be predicted from the observed underlying patterns of genetic relatedness (Dubin et al., 2015). Genetic variation could also not explain the association between epigenetic variation and phenotypic variation (leaf, petiole and functional traits) in natural populations of the perennial herb *Helleborus foetidus* (Schulz et al., 2014; Foust et al., 2016; Gugger et al., 2016), and in clonal populations of the introduced clonal herb *Hydrocotyle vulgaris* (Medrano et al., 2014). In agreement, the correlation between methylome variation and photosynthetic performance in our study could not be predicted from the underlying genetic variation. This suggests that differences in performance among ramets in clonal plants can be explained not exclusively by somatic mutations (Wang et al., 2020) but at least partly by independent differences in their methylome. Independent from genetic variation was also the methylome shift in the experimental samples that provides potential for temporally stable phenotypic change at time scales unattainable by somatic mutations. Particularly across mitotically grown generations, epigenetic patterns can be expected to be more faithfully inherited than across sexual generations, because clonal growth circumvents epigenetic reprogramming during meiosis and embryogenesis (Santelices et al., 2018; Simberloff and Leppanen, 2019; Yu et al., 2020). Thus, our results provide a first indication that DNA methylation variation provides a layer of ecologically relevant phenotypic variation that is independent from genetic variation in clonal seagrass meadows.

### Methylome Shift Over the Course of the Heat-Stress Experiment

Methylation profiles changed over the course of the heat stress experiment with a clear shift from control to stress and recovery profiles (**Figure 7**). Interestingly, methylation profiles were highly similar between the stress and recovery phase as shown by the overlap of the 95%-confidence intervals around the group means (**Figure 7**, **Supplementary Figure S3**), and the low number of differentially expressed sites between stress and recovery samples (**Figures 8C, F**, **Supplementary Table S11**). Nevertheless, recovery profiles showed an apparently stronger divergence from the control profiles (**Figures 8B, E**) than the stress profiles did (**Figures 8A, D**). This shift in methylation profiles may be due to different processes that likely acted in parallel.

A first explanation is that part of the recorded methylome shift was likely induced by heat stress, given that the recovered shoots showed hyper-methylation and, thus, potentially constitutive upregulation (Zhang et al., 2006; Schmitz et al., 2013; Yang et al., 2014; Dubin et al., 2015; Niederhuth and Schmitz, 2017) of genes involved in the functions of catabolism of misfolded proteins (GO:0006515) (**Supplementary Table S12**). This suggests increased investment in the breakdown of heat-denatured proteins, a common response to heat-stress (Feder and Hofmann, 2002). This function was also hyper-methylated in control samples of highest photosynthetic performance (**Supplementary Table S14**), that also recovered better from the stress (although some samples had died, **Figure 2**). Heat-responsive DNA methylation changes in plants appear not to show a consistent trend across different species, and little is yet known about their functional role (Liu et al., 2015). In *Brassica rapa*, heat-responsive DNA methylation was shown to be associated with differential expression of genes involved in RNA metabolic processing, and in heat stress signal transduction (Liu et al., 2018). Stress signal transduction may also have been affected by the experimental methylation changes in *Z*. *marina* as enriched biological processes in recovered shoots included the regulation of transmembrane transport of ions and protons (GO:0006811, GO:0055085, GO:0006810, GO:0034220, GO:0006812, GO:0098655, GO:0098660, GO:0015672, GO:0099132, GO:0015991, GO:0098662, GO:0099131, GO:0015988, GO:1902600), and neurotransmitter levels (GO:0042133, GO0042135, GO:0001505) (**Supplementary Table S12**). Experimental removal of DNA methylation, e.g. using zebularine or 5-Azacytidine (Griffin et al., 2016), or the targeted change of methylation patterns, e.g. *via* CRISPR (Xu et al., 2016), will ultimately allow us to identify the relationship between methylation changes under stress and adaptive phenotypic changes.

However, an alternative explanation for the shift in methylome profiles over the course of the experiment is the continued acclimation of seagrass shoots to laboratory conditions. In order to correlate methylome differences with photosynthetic performance differences between the epigenotypes, we had to choose a longitudinal sampling design in which we followed single shoots/epigenotypes over the course of the experiment. To reduce the effect of acclimation on methylome profiles, we allowed for a 2-week acclimation period before the start of the experiment, but can not rule out that the methylome shift from field to lab conditions (**Figure 4**) was completed after this time period. Moreover, although we had planted the shoots in soil from the sampling site and observed continuous growth of the shoots, we cannot exclude the possibility that the dilution of natural seawater with freshwater may have resulted in lower nutrient contents as compared with field conditions, which may have induced a stress factor in addition to the applied heat. Most likely, heat-stress, acclimation, and nutrient depletion have contributed in parallel to the recorded methylome shift and can not be disentangled in this study. Thus, future studies need to run a cross-sectional sampling design with unstressed samples as control treatment in parallel to a longitudinal sampling design that traces methylome changes in single epigenotypes, in order to distinguish methylome changes induced by transplantation to laboratory conditions from such induced exclusively by the applied heat-stress.

If the recorded methylation shift over the experiment has been primarily a response to the applied heat-stress, the similarity in methylation profiles between stress and recovery phases can be interpreted as the formation of an epigenetic stress memory (Crisp et al., 2016). Continued divergence of methylation profiles from stress to recovery conditions (**Figures 8B, D**) agrees with increasing divergence of gene transcription profiles from heat-stress to recovery conditions in a Danish *Z*. *marina* population (Franssen et al., 2011). We speculate that gene expression changes, and other molecular mechanisms involved in the heat-stress response, could have triggered additional methylation changes after the stress was removed (e.g. Secco et al., 2015). A stress-memory lasting longer than 5 weeks, would be long enough to potentially heat-harden the same generation of previously exposed shoots. In agriculture, hardening/priming of seeds is a long-standing practice to enhance crop resistance to environmental challenges, including hot, cold, dry, or saline conditions, or pathogen infections (Ibrahim, 2016; Wojtyla et al., 2016; Pawar and Laware, 2018). Priming refers to the plants’ ability to acquire a stress memory that enhances performance under second stress exposure by responding faster, stronger, or in response to a lower threshold as compared with naïve plants (Balmer et al., 2015; Lämke and Bäurle, 2017). Molecular mechanisms involved in forming a stress memory include stalled RNA polymerase II, storage of chemical signaling factors, accumulation and phosphorylation of transcription factors, and epigenetic mechanisms such as microRNAs, histone modifications, and DNA methylation (Iwasaki and Paszkowski, 2014; Crisp et al., 2016; Hilker et al., 2016; Gallusci et al., 2017; Lämke and Bäurle, 2017). Heat-priming has only very recently been described in seagrasses (Nguyen et al., 2020). *Zostera muelleri* and *Posidonia australis* both performed better under a second heat-wave, in terms of photosynthetic capacity, leaf growth, and chlorophyll *a* content, when they had been previously exposed to a first heat-wave as compared with naïve controls (Nguyen et al., 2020). This could explain why no mortality was reported for the seagrass *Posidoinia oceanica* after intense and long-lasting heat-waves in 2012, 2015, and 2017 (Darmaraki et al., 2019), although it had suffered high mortality rates after the 2006 heatwave ((Marbà and Duarte, 2010), as discussed in Nguyen et al. (2020). However, whether the experimental shift in methylation patterns in *Z*. *marina* may be involved in heat-priming can only be answered *via* exposure to a second heat-wave.

### Conclusions and Perspectives

Our study suggests that DNA methylation is functionally relevant for photosynthetic performance, independent from underlying somatic mutations. In seagrass meadows composed of several genotypes, stress resilience, growth, and associated invertebrate species diversity is enhanced by genotypic variation (Hughes and Stachowicz, 2004; Reusch et al., 2005; Ehlers et al., 2008). In clonal meadows, epigenetic variation may play a similar role in the potential to secure function and resilience not only of *Z*. *marina* plants, but also of the entire associated ecosystem.

Due to anthropogenic stressors, nearly one-third of global seagrass area has disappeared over the last 100 years, and the rate of loss accelerated from ca. 1% yr^−1^ before 1940 to 7% yr^−1^ since 1990 (Waycott et al., 2009). At the same time, rising temperatures open up new thermally suitable habitat in the Arctic (Krause-Jensen and Duarte, 2014). How fast and far warm-temperate and subarctic range edges will move polewards depends on the ability of seagrass to rapidly acclimate and adapt to rising temperatures and other environmental changes (Duarte et al., 2018). Thus, future studies are needed to assess the adaptive value and transgenerational stability of the epigenetic stress response, and to compare the ability to build up epigenetic variation between seagrass meadows composed of a single or multiple clones, as well as between range center *versus* edge populations.

The functional role of methylation variation in plant genets is not only of fundamental interest but also of applied interest for management programs of clonal organisms designed to assess evolutionary potential and population stability, and to minimize the loss of biodiversity. Our results can be relevant for restoration of seagrass ecosystems that largely depends on the success of replanted shoots to overcome natural variability/stress (Suykerbuyk et al., 2016; van Katwijk et al., 2016). Given that 40% of all plant species can reproduce clonally (Tiffney and Niklas, 1985), our findings are further important to other fields, such as invasion biology and crop breeding strategies (Bilichak and Kovalchuk, 2016) of clonal plants.

## Data Availability Statement

The datasets presented in this study can be found in online repositories. The names of the repository/repositories and accession number(s) can be found in the article/**Supplementary Material**.

## Author Contributions

GH (project leader) and AJ were planning the project and designing the experiments. AJ, CB, IS, and GH collected the shoots. AJ analyzed the data and wrote the manuscript. AJ, MK, AD, JC, and IS performed the DNA extraction, library preparation, and sequencing. SA-H, JO, and YP were involved in data interpretation. All authors contributed to the article and approved the submitted version.

## Funding

This work was supported by the Norwegian Research Council (Havkyst project 243916), the Åbo Akademi University Foundation sr to CB, and a personal research talents grant from Nord University to AJ. Open access publication fees were covered by Nord University’s Opean Access fund.

## Conflict of Interest

The authors declare that the research was conducted in the absence of any commercial or financial relationships that could be construed as a potential conflict of interest.

## Footnotes

1 http://www.bioinformatics.babraham.ac.uk/projects/fastqc/

2 https://www.bioinformatics.babraham.ac.uk/projects/trim\_galore/

3 https://www.bioinformatics.babraham.ac.uk/projects/trim\_galore/

4 http://www.bioinformatics.babraham.ac.uk/projects/fastqc/

5 http://marinetics.org/2017/04/11/REdigestions.html

6 http://www.repeatmasker.org/RepeatModeler/
