# Supplementary material for: The Seagrass Methylome Is Associated With Variation in Photosynthetic Performance Among Clonal Shoots
Source: Front Plant Sci. 2020 Sep 4;11:571646. doi: 10.3389/fpls.2020.571646 (PMC7498905; doi:10.3389/fpls.2020.571646)
Supplement: Supplementary file 1 [file DataSheet_1.pdf]

Supplementary Material

1 Supplementary Figures

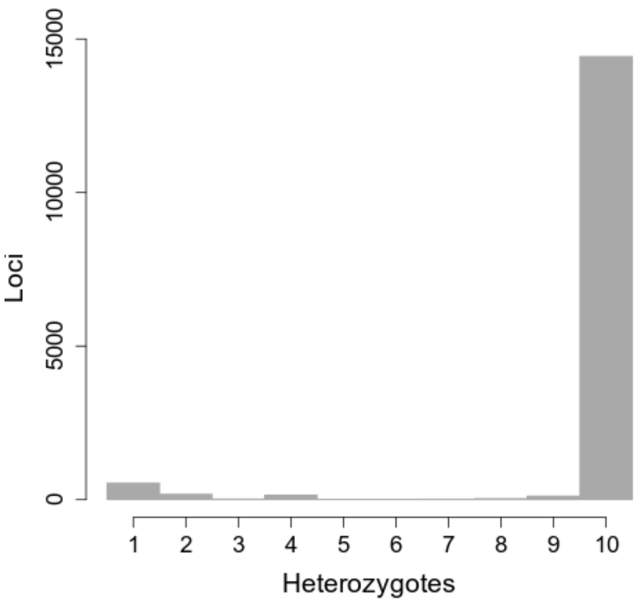

Supplementary Figure S1 Number of heat-stressed *Zostera marina* shoots sharing the same heterozygous state for 15,508 biallelic SNPs. The dominant peak of loci where all shoots are identically heterozygous suggests that all ten shoots belong to the same genet, and inherited the heterozygous state.

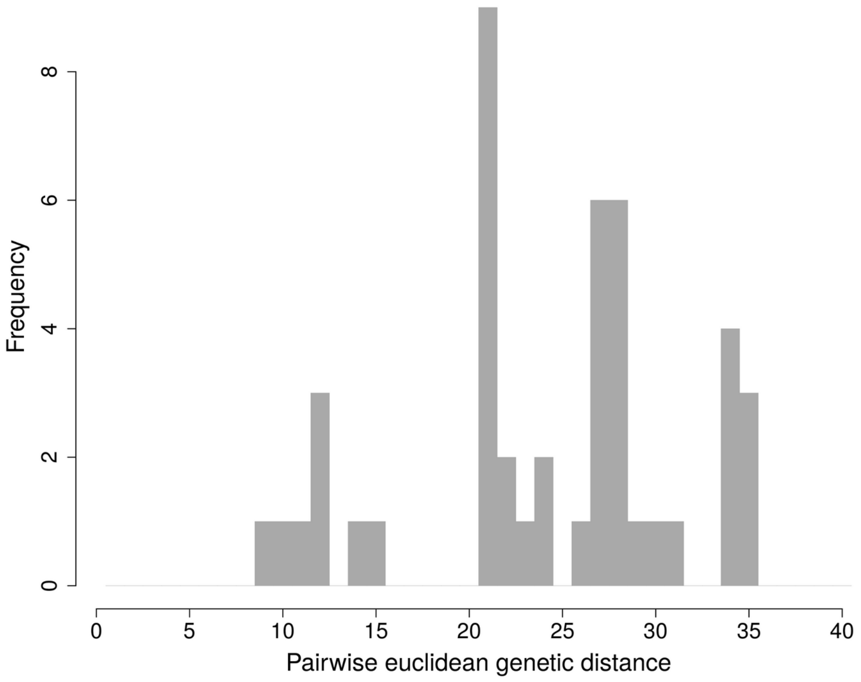

Supplementary Figure S2 Frequency distribution of pairwise euclidean distances among 10 heat-stressed *Zostera marina* shoots based on 1,079 SNPs resulting from somatic mutations.

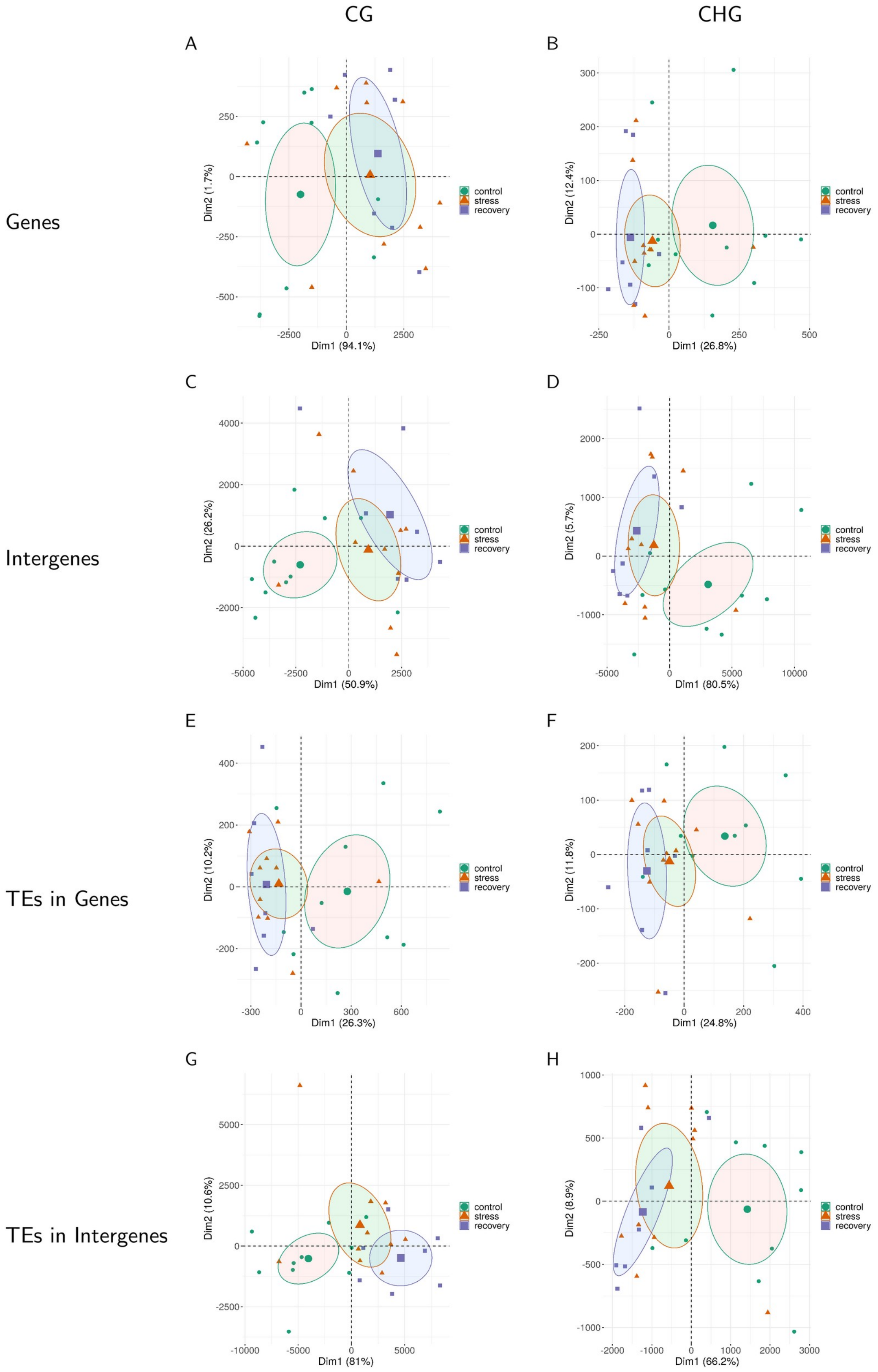

Supplementary Figure S3 Methylation patterns in shoots of a *Zostera marina* clone changed in response to heat stress and did not return to pre-stress patterns after a 5-week recovery period. This applies for all sequence contexts, including genes, intergenes, and transposable elments (TEs) in genes or intergenes in either CG or CHG sequence contexts The samples are plotted along the first

two principle components (Dim) based on methylation profiles across all sequence contexts. Circles represent 95% confidence intervals around group means. Bracketed numbers represent the percentage of explained variation.

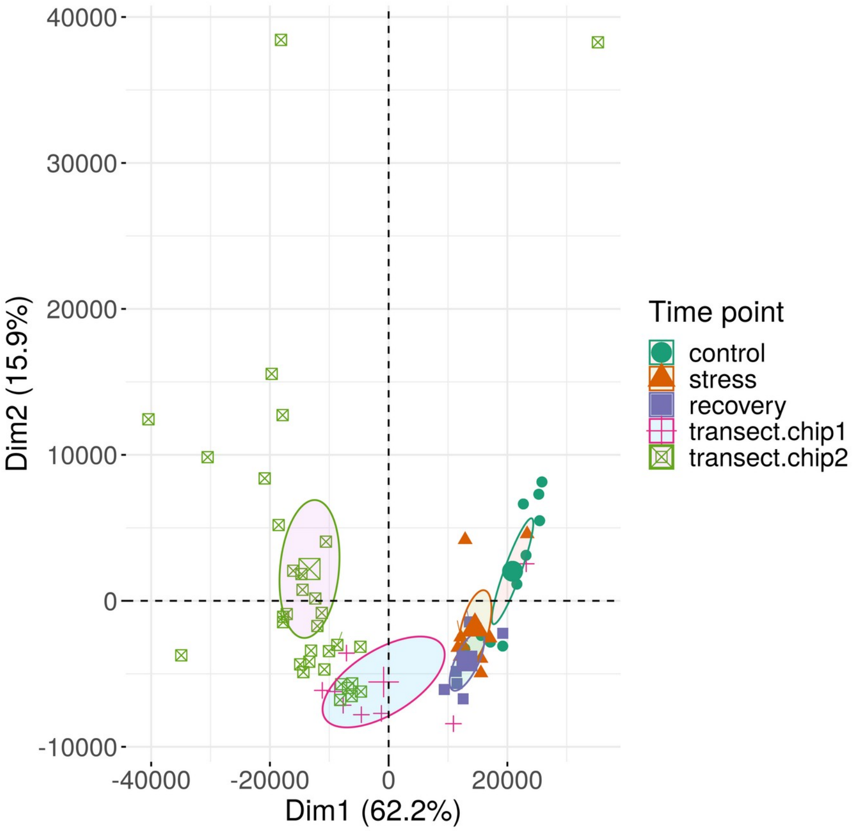

Supplementary Figure 4 Methylation variation of field transect samples, separated by sequencing chip (transect.chip1, transect.chip2), and of experimental samples (all run together on a third sequencing chip) of *Zostera marina* along the first two principal components. The samples are plotted along the first two principle components (Dim) based on methylation profiles across all sequence contexts. Circles represent 95% confidence intervals around group means. Bracketed numbers represent the percentage of explained variation.

2 Supplementary Tables

Supplementary Table S1 Seagrass samples that were taken along a ca. 250 m transect. Listed are the location and IDs of the 42 samples that were sequenced directly after sampling, and of the 10 samples that took part in the heat stress experiment. Note that the sample number is a consecutive number

Supplementary Table S2 Number of raw and filtered (high-quality) reads of genomic libraries prepared for each of the ten heat-stressed samples according to the TruSeq DNA PCR-Free (Illumina) protocol, and sequenced on one Illumina HiSeq 3/4000 lane (2x150bp).

Supplementary Table S3 Number of sequenced reads, trimmed (high-quality) reads, mapped, and uniquely mapped reads for each sample. The flow-cell each sample was sequenced on is listed.

Supplementary Table S4 Number of 628,255 *in silico* predicted MethylRAD tags annotated to the different sequence contexts (Gene/Intergene region, Transposable element, CG or CHG context).

Supplementary Table S5 Number of methylated CG and CHG sites in genes, intergenic regions, and transposable elements, listed for each transect sample. The minimum, mean, and maximum number of regions that were differently methylated between the samples are listed in the last two columns. Comparisons with sample 27 are interclonal comparisons, the other intraclonal.

Supplementary Table S6 Microsatellite raw data. Microsatellite fragment lengths for 7 markers (2 columns = 2 alleles per marker) for each sample (ID in the first column).

Supplementary Table S7 Mantel test results (p-values adjusted by Benjamini-Hochberg correction and Pearson's Correlation Coefficient R) for correlations between epigenetic and geographic distance at each sequence context.

Supplementary Table S8 Photosynthetic performance measures. Mean values and standard errors of PiABS for each of the 10 shoots at all three sampling points in the heat stress experiment.

Supplementary Table S9 Raw PAM data. For each sample, specified by its ID, two measurements from two leafs were taken control, stress, and recovery conditions. The following parameters are provided: Bckg: Background; F_0: $F_{50\mu s}$, fluorescence intensity at 50 µs; F_J = fluorescence intensity at J-step (at 2 ms); Fi: fluorescence intensity at i-step (at 60 ms); F_M: maximal fluorescence intensity; F_V: F_M - F_0 (maximal variable fluorescence); V_J: (F_J - F_0) / (F_M - F_0); V_i:(F_i - F_0) / (F_M - F_0); F_M / F_0; F_V / F_0; F_v / F_M; M_0 or $(dV/dt)_0$: TR_0 / RC - ET_0 / RC = 4 (F_{300} - F_0) / (F_M - F_0); Area: Area between fluorescence curve and F_M (background subtracted); Fix Area: Area below the fluorescence curve between $F_{40\mu s}$ and F_{1s} (background subtracted); S_M: Area / (F_M - F_0) (multiple turn-over); S_S: the smallest S_M turn-over (single turn-over); N: S_M . M_0 . (1 / V_J) turn-over number Q_A; Phi_P_0: 1 – (F_0 / F_M) (or F_V / F_M); Psi_$_0$: 1 – V_J; Phi_E_0: (1 – (F_0 / F_M)) . Psi_$_0$; Phi_D_0: 1 – Phi_P_0 – (F_0 / F_M); Phi_Pav = Phi_P_0 (S_M / t_{FM}) with t_{FM}: time to reach F_M (in ms); ABS / RC: M_0 . (1 / V_J) . (1 / Phi_P_0); TR_0 / RC: M_0 . (1 / V_J); ET_0 / RC: M_0 . (1 / V_J) . Psi_$_0$; DI_0 / RC:(ABS / RC) – (TR_0 / RC).

Supplementary Table S10 Correlation matrix of photosynthetic performance (PiABS) differences (columns D-F) or of genetic distances (column E) among clonal *Zostera marina* shoots with epigenetic distances (rows 3-26) or with performance differences (rows 27-29) at control, stress, or recovery conditions. The table mirrors Figure 6 and provides Pearson product-moment correlation coefficients *R* and Benjamini-Hochberg adjusted *p*-values for all tested correlations. All correlations between photosynthetic performance differences and epigenetic distances were controlled for genetic distances.

Supplementary Table S11 Number of differentially methylated sites (column C, D), and total number of methylated sites (methylated in at least one of the samples, column E,F) between control vs. stress, control vs. recovery, and stress vs. recovery samples listed for each sequence context.

Supplementary Table S12 Enriched biological processes showing differential methylation in gene bodies between control and recovery samples. The Supplementary Table lists the direction of methylation (hyper- or hypomethylated in comparison with control samples); whether the methylation site falls within a transposable element; the sequence context (CG or CHG); the GO term ID; description; frequency (proportion of this GO term in the underlying *Arabidopsis thaliana* protein annotation database; log10 p-value of the enrichment test; the term's uniqueness; dispensability (the semantic similarity threshold at which the term was removed from the list and

assigned to a cluster); the representative GO term; and a boolean indicating whether the ID stands alone or was assigned to a cluster (eliminated).

Supplementary Table S13 Differentially methylated sites between two samples of highest (79.1 and 13.2 for control conditions, 79.1 and 17.1 for recovery conditions) and two samples of lowest photosynthetic performance (63.1 and 59.2 for control conditions, 57.1 and 59.2 for recovery conditions). The Supplementary Table contains the following columns: Id, unique MethylRAD tag identifier (location in the genome v2.1); sampleName, raw counts per sample; norm.sampleName, rounded normalized counts per sample; baseMean, base mean over all samples; lowperformance and highperformance, means (rounded) of normalized counts of the biological conditions; FoldChange, fold change of expression; log2FoldChange, estimated by the GLM model. It reflects the differential expression between Test and Ref. If this value is around 0 (the feature methylation is similar in both conditions), positive (the feature is more methylated in the samples of high photosynthetic performance), negative, (the feature is less methylated in the samples of high photosynthetic performance); pvalue, raw p-value from the statistical test; padj, p-value adjusted by Benjamini-Hochberg correction; tagwise.dispersion, dispersion parameter estimated from feature counts; trended.dispersion, dispersion parameter estimated with splines; GeneID, gene identifier based on the /Zostera marina/ genome annotation v2.1; Gene Name; GO Terms.

Supplementary Table S14 Enriched biological processes with increased methylation in control samples of high performance. The Supplementary Table lists the GO term ID; description; frequency (proportion of this GO term in the underlying *Arabidopsis thaliana* protein annotation database; log10 p-value of the enrichment test; the term's uniqueness; dispensability (the semantic similarity threshold at which the term was removed from the list and assigned to a cluster); the representative GO term; and a boolean indicating whether the ID stands alone or was assigned to a cluster (eliminated).

3 Supplementary Files

Supplementary File S1 Biallelic Single Nucleotide Polymorphisms (15,508) that differ from the reference genome or among the 10 heat-stressed samples of the seagrass *Zostera marina*.

Supplementary File S2 Biallelic Single Nucleotide Polymorphisms (1,079) that differ among the 10 heat-stressed samples of the seagrass *Zostera marina.*

Supplementary File S3 Adapters and primers used to prepare MethylRAD libraries.

Supplementary File S4 Annotated reads-per-million for 42 transect shoots based on the Z. *marina* genome annotation v2.1 (GenBank Accession nos. LFYR00000000) listing the geneID, location on the Scaffold, reads-per-million for all samples, region (genic or intergenic), TE (whether it falls within a transposable elment), Name (gene name), GOs (associated gene ontology terms), and SequenceContexts (CG or CHG). The data are openly available in figshare at 10.6084/m9.figshare.9929132.

Supplementary File S5 Annotated reads-per-million for experimental samples (control, stress, and recovery) based on the *Z. marina* genome annotation v2.1 (GenBank Accession nos. LFYR00000000) listing the geneID, location on the Scaffold, reads-per-million for all samples, region (genic or intergenic), TE (whether it falls within a transposable elment), Name (gene name), GOs (associated gene ontology terms), and SequenceContexts (CG or CHG). The data are openly available in figshare at 10.6084/m9.figshare.9929171.
